# 3-Hy­droxy-2-meth­oxy­benzamide

**DOI:** 10.1107/S1600536812047769

**Published:** 2012-11-30

**Authors:** Sabine Wilbrand, Christian Neis, Kaspar Hegetschweiler

**Affiliations:** a Fachrichtung Chemie, Universität des Saarlandes, Postfach 151150, D-66041 Saarbrücken, Germany

## Abstract

The crystal structure of the title compound, C_8_H_9_NO_3_, features centrosymmetric dimers with two amide groups inter­connected by a pair of almost linear N—H⋯O hydrogen bonds. Through inter­molecular O—H⋯O inter­actions between phenolic hy­droxy groups and carbonyl O atoms, these dimers are assembled into undulating hydrogen-bonded layers parallel to the [101] plane. Additionally, the *anti*-H(—N) atom of the primary amide group forms an intra­molecular hydrogen bond to the O atom of the meth­oxy group. The amide group froms a dihedral angle of 12.6 (1)° with the phenyl ring.

## Related literature

Hydrogen-bonding packing patterns of primary amides are discussed by Eccles *et al.* (2011 ▶) and McMahon *et al.* (2005 ▶). A description of the Cambridge Crystallographic Database is given by Allen (2002 ▶). The question of the occurrence of very bent, intra­molecular C—H⋯O hydrogen bonds has been discussed by Desiraju (1996 ▶).
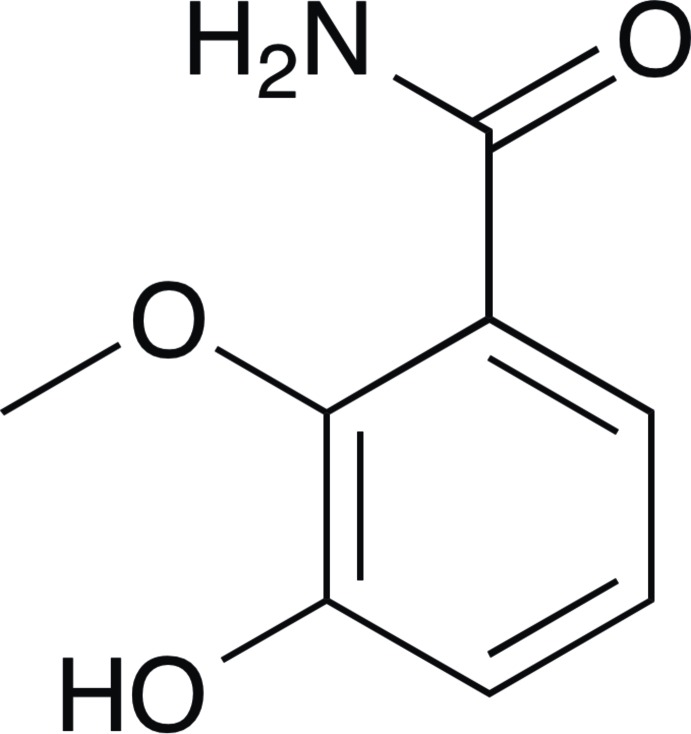


## Experimental

### 

#### Crystal data

C_8_H_9_NO_3_
*M* *_r_* = 167.16
Monoclinic, 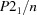

*a* = 5.6293 (2) Å
*b* = 10.1826 (4) Å
*c* = 13.2402 (5) Å
β = 92.750 (1)°
*V* = 758.07 (5) Å^3^
*Z* = 4
Mo *K*α radiation
μ = 0.11 mm^−1^
*T* = 110 K
0.49 × 0.18 × 0.14 mm

#### Data collection

Bruker APEXII KappaCCD diffractometer
Absorption correction: multi-scan (*SADABS*; Bruker, 2010 ▶) *T*_min_ = 0.947, *T*_max_ = 0.984
7200 measured reflections
1484 independent reflections
1325 reflections with *I* > 2σ(*I*)
*R*_int_ = 0.022

#### Refinement

*R*[*F* ^2^ > 2σ(*F* ^2^)] = 0.030
*wR*(*F* ^2^) = 0.083
*S* = 1.06
1484 reflections
122 parameters
H atoms treated by a mixture of independent and constrained refinement
Δρ_max_ = 0.27 e Å^−3^
Δρ_min_ = −0.19 e Å^−3^

### 

Data collection: *APEX2* (Bruker, 2010 ▶); cell refinement: *SAINT* (Bruker, 2010 ▶); data reduction: *SAINT*; program(s) used to solve structure: *SHELXS97* (Sheldrick, 2008 ▶); program(s) used to refine structure: *SHELXL97* (Sheldrick, 2008 ▶); molecular graphics: *DIAMOND* (Brandenburg, 2012 ▶); software used to prepare material for publication: *SHELXL97*.

## Supplementary Material

Crystal structure: contains datablock(s) global, I. DOI: 10.1107/S1600536812047769/ld2083sup1.cif

Structure factors: contains datablock(s) I. DOI: 10.1107/S1600536812047769/ld2083Isup2.hkl

Supplementary material file. DOI: 10.1107/S1600536812047769/ld2083Isup3.cml

Additional supplementary materials:  crystallographic information; 3D view; checkCIF report

## Figures and Tables

**Table 1:** Hydrogen-bond geometry (Å, °)

*D*—H⋯*A*	*D*—H	H⋯*A*	*D*⋯*A*	*D*—H⋯*A*
N1—H1*A*⋯O3^i^	0.901 (17)	2.056 (17)	2.9547 (13)	175.3 (14)
N1—H1*B*⋯O1	0.887 (17)	1.977 (17)	2.6789 (13)	135.0 (14)
O2—H2⋯O3^ii^	0.88 (2)	1.83 (2)	2.6895 (12)	165.5 (17)

Symmetry codes: (i) 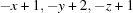; (ii) 
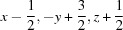
.

**Fig. 1.**
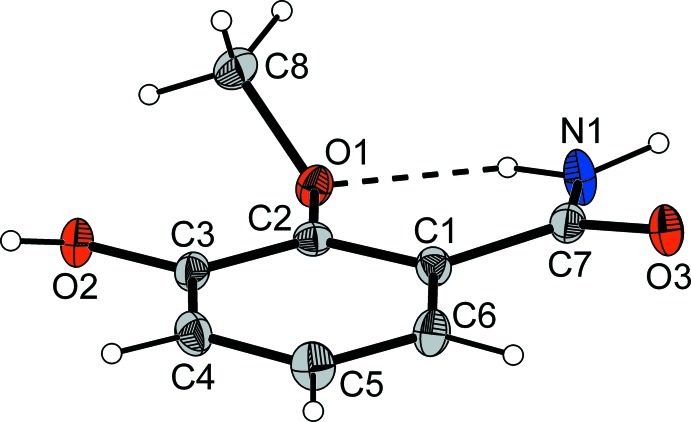
Ellipsoid plot (50% probability level) and numbering scheme of the title compound.

**Fig. 2.**
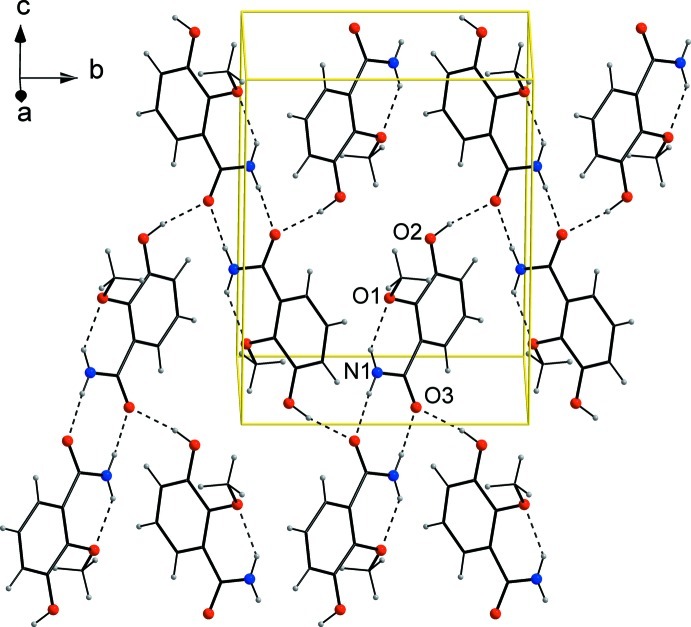
A section of the hydrogen-bonding network.

## supplementary crystallographic information

### Comment

The formation of hydrogen-bonded head-to-head dimers is well established for primary amides. A comprehensive search (McMahon *et al.*, 2005) in the Cambridge Structural Database (Allen, 2002) revealed that 84% of the primary amides form such dimers. In addition, 14% form a catemeric (infinite chain) structure. It has recently been demonstrated that the dimers may, however, readily be disrupted in the presence of additional hydrogen-accepting functional groups (Eccles *et al.*, 2011). Although the title compound possesses two such additional groups, the head-to-head dimer formation in the crystal structure is retained.

In the title compound, the two plains defined by the phenyl ring (C1 - C6) and the amide group (C1, C7, O3, N1) are slightly tilted against each other with an angle of 12.6 (1)°. Since the amide, methoxy and hydroxy group are arranged in a consecutive 1,2,3-arrangement, the ether oxygen atom of the methoxy group would be capable to accept either the O—H or N—H proton from the two adjacent moieties, forming a five- or six-membered ring structure, respectively. It is generally accepted that the latter is favoured, and this has indeed been observed. Owing to the steric demands of the 1,2,3-arrangement, the methoxy methyl group is significantly displaced from the aromatic plain, avoiding congestion: The distance of the methyl carbon atom to the aromatic mean plane is 0.819 (2) Å. This is a common feature for 3-alkoxy- or 3-hydroxy-2-methoxybenzamides. A search in the Cambridge Structural Database revealed a total of 46 entries for this structure type, and all of them show a similar displacement. An unexpected feature in the title compound is, however, the orientation of the three hydrogen atoms of the methyl group with an H8C—C8—O1—C2 torsional angle of 24.2 °. This value approaches an eclipsed rather than a staggered conformation. In the final refinement, these hydrogen atoms were treated as rigid group which was, however, allowed to rotate freely. It is clear that the effect under discussion is related to a rather small amount of electron density. However, a corresponding refinement, where the three hydrogen atoms were forced to adopt a staggered orientation, resulted in an increase of wR2(all) from 8.28 to 13.75%! Moreover, full refinement of the positional parameters revealed again the previously obtained, non-staggered arrangement (H8C—C8—O1—C2 torsional angle = 25.4 °, wR2(all) = 8.12%). Notably, in the above-mentioned 46 structures found in our CSD search, only three of them (LUDXEN, IPUQEP, SIGKIC) exhibit a similar deviation from a staggered orientation. Obviously, attractive and repulsive interactions account for this particular structure, and it is tempting to interpret the H8A···O2 distance of 2.49 Å in terms of some intramolecular C—H···O hydrogen bonding. However, the small C8—H8A···O2 angle of 98 ° indicates that such an interaction would be - if at all - rather weak (Desiraju, 1996).

### Experimental

2,3-Bis(benzyloxy)benzoic acid was obtained from 2,3-dihydroxybenzoic acid (K_2_CO_3_, benzyl bromide). It was converted into the corresponding amide *via* the acid chloride using thionyl chloride and subsequently an aqueous ammonia solution. The benzyl groups were then removed (ammonium formiate, Pd/C), and the resulting 2,3-dihydroxybenzamide was methylated in DMF using iodomethane and potassium bicarbonate. Some by-products were removed by chromatographic methods (SiO_2_, hexane / Et_2_O), and the title compound was obtained as a colourless solid. ^1^H-NMR (DMSO-d_6_): δ (p.p.m.) = 3.76 (s, 3H), 6.95 (m, 2H), 7.06 (dd, 1H), 7.43 (NH), 7.65 (NH). ^13^C-NMR (DMSO-d_6_): δ (p.p.m.) = 60.7, 118.8, 119.7, 123.9, 129.5, 145.6, 150.4, 167.3. Single crystals were grown from Et_2_O.

### Refinement

The 3-hydroxy-2-methoxybenzamide molecule could be refined without problems, and its hydrogen atoms could all be located. The H(—C) positions were calculated (riding model). The methyl group was allowed to rotate freely. All positional parameters of the O- and N-bonded H-atoms were refined using variable isotropic displacement parameters.

### Crystal data

**Table:**

C_8_H_9_NO_3_	*F*(000) = 352
*M**_r_* = 167.16	*D*_x_ = 1.465 Mg m^−^^3^
Monoclinic, *P*2_1_/*n*	Mo *K*α radiation, λ = 0.71073 Å
*a* = 5.6293 (2) Å	Cell parameters from 3853 reflections
*b* = 10.1826 (4) Å	θ = 2.5–32.5°
*c* = 13.2402 (5) Å	µ = 0.11 mm^−^^1^
β = 92.750 (1)°	*T* = 110 K
*V* = 758.07 (5) Å^3^	Prism, colourless
*Z* = 4	0.49 × 0.18 × 0.14 mm

### Data collection

**Table:**

Bruker APEXII KappaCCD diffractometer	1484 independent reflections
Radiation source: fine-focus sealed tube	1325 reflections with *I* > 2σ(*I*)
Graphite monochromator	*R*_int_ = 0.022
phi and ω scans	θ_max_ = 26.0°, θ_min_ = 2.5°
Absorption correction: multi-scan (*SADABS*; Bruker, 2010)	*h* = −6→6
*T*_min_ = 0.947, *T*_max_ = 0.984	*k* = −12→12
7200 measured reflections	*l* = −15→16

### Refinement

**Table:**

Refinement on *F*^2^	Primary atom site location: structure-invariant direct methods
Least-squares matrix: full	Secondary atom site location: difference Fourier map
*R*[*F*^2^ > 2σ(*F*^2^)] = 0.030	Hydrogen site location: inferred from neighbouring sites
*wR*(*F*^2^) = 0.083	H atoms treated by a mixture of independent and constrained refinement
*S* = 1.06	*w* = 1/[σ^2^(*F*_o_^2^) + (0.0421*P*)^2^ + 0.2806*P*] where *P* = (*F*_o_^2^ + 2*F*_c_^2^)/3
1484 reflections	(Δ/σ)_max_ = 0.001
122 parameters	Δρ_max_ = 0.27 e Å^−^^3^
0 restraints	Δρ_min_ = −0.19 e Å^−^^3^

### Special details

**Table:**

Geometry. All e.s.d.'s (except the e.s.d. in the dihedral angle between two l.s. planes) are estimated using the full covariance matrix. The cell e.s.d.'s are taken into account individually in the estimation of e.s.d.'s in distances, angles and torsion angles; correlations between e.s.d.'s in cell parameters are only used when they are defined by crystal symmetry. An approximate (isotropic) treatment of cell e.s.d.'s is used for estimating e.s.d.'s involving l.s. planes.
Refinement. Refinement of *F*^2^ against ALL reflections. The weighted *R*-factor *wR* and goodness of fit *S* are based on *F*^2^, conventional *R*-factors *R* are based on *F*, with *F* set to zero for negative *F*^2^. The threshold expression of *F*^2^ > σ(*F*^2^) is used only for calculating *R*-factors(gt) *etc*. and is not relevant to the choice of reflections for refinement. *R*-factors based on *F*^2^ are statistically about twice as large as those based on *F*, and *R*- factors based on ALL data will be even larger.

### Fractional atomic coordinates and isotropic or equivalent isotropic displacement parameters (Å^2^)

**Table:**

	*x*	*y*	*z*	*U*_iso_*/*U*_eq_	
O1	0.17410 (14)	0.98464 (8)	0.80141 (6)	0.0160 (2)	
O2	−0.12850 (15)	0.83936 (9)	0.91393 (6)	0.0192 (2)	
H2	−0.195 (3)	0.7717 (19)	0.9432 (13)	0.046 (5)*	
O3	0.23900 (15)	0.88839 (8)	0.49736 (6)	0.0194 (2)	
N1	0.35604 (19)	1.02761 (11)	0.62107 (8)	0.0207 (3)	
H1A	0.474 (3)	1.0570 (15)	0.5835 (12)	0.031 (4)*	
H1B	0.346 (3)	1.0515 (16)	0.6851 (13)	0.031 (4)*	
C1	0.0386 (2)	0.87000 (11)	0.65116 (9)	0.0149 (3)	
C2	0.03195 (19)	0.88969 (11)	0.75592 (8)	0.0137 (3)	
C3	−0.1284 (2)	0.81706 (11)	0.81214 (9)	0.0156 (3)	
C4	−0.2821 (2)	0.72817 (12)	0.76327 (9)	0.0182 (3)	
H4	−0.3876	0.6775	0.8014	0.022*	
C5	−0.2824 (2)	0.71313 (12)	0.65908 (9)	0.0203 (3)	
H5	−0.3918	0.6546	0.6258	0.024*	
C6	−0.1234 (2)	0.78327 (12)	0.60378 (9)	0.0185 (3)	
H6	−0.1243	0.7723	0.5325	0.022*	
C7	0.2192 (2)	0.93056 (11)	0.58521 (9)	0.0158 (3)	
C8	0.3210 (2)	0.94758 (13)	0.88935 (9)	0.0213 (3)	
H8A	0.2392	0.9700	0.9507	0.032*	
H8B	0.4727	0.9948	0.8890	0.032*	
H8C	0.3508	0.8528	0.8879	0.032*	

### Atomic displacement parameters (Å^2^)

**Table:**

	*U*^11^	*U*^22^	*U*^33^	*U*^12^	*U*^13^	*U*^23^
O1	0.0186 (4)	0.0168 (4)	0.0125 (4)	−0.0020 (3)	−0.0006 (3)	−0.0004 (3)
O2	0.0235 (5)	0.0217 (5)	0.0125 (4)	−0.0022 (4)	0.0045 (3)	0.0010 (3)
O3	0.0241 (5)	0.0209 (5)	0.0138 (4)	−0.0026 (3)	0.0063 (3)	−0.0024 (3)
N1	0.0247 (6)	0.0232 (6)	0.0150 (5)	−0.0082 (4)	0.0083 (4)	−0.0027 (4)
C1	0.0167 (6)	0.0138 (6)	0.0146 (6)	0.0017 (4)	0.0033 (4)	0.0007 (4)
C2	0.0137 (5)	0.0121 (5)	0.0152 (6)	0.0019 (4)	0.0002 (4)	−0.0008 (4)
C3	0.0183 (6)	0.0160 (6)	0.0126 (5)	0.0036 (5)	0.0026 (4)	0.0010 (4)
C4	0.0185 (6)	0.0172 (6)	0.0193 (6)	−0.0015 (5)	0.0053 (5)	0.0025 (5)
C5	0.0213 (6)	0.0185 (6)	0.0210 (6)	−0.0052 (5)	0.0012 (5)	−0.0022 (5)
C6	0.0231 (6)	0.0196 (6)	0.0128 (6)	−0.0014 (5)	0.0021 (5)	−0.0018 (5)
C7	0.0180 (6)	0.0156 (6)	0.0138 (6)	0.0018 (4)	0.0024 (4)	0.0010 (5)
C8	0.0192 (6)	0.0277 (7)	0.0165 (6)	−0.0008 (5)	−0.0035 (5)	0.0012 (5)

### Geometric parameters (Å, º)

**Table:**

O1—C2	1.3753 (14)	C2—C3	1.4074 (16)
O1—C8	1.4456 (14)	C3—C4	1.3903 (17)
O2—C3	1.3668 (14)	C4—C5	1.3879 (17)
O2—H2	0.88 (2)	C4—H4	0.9500
O3—C7	1.2499 (14)	C5—C6	1.3822 (17)
N1—C7	1.3267 (16)	C5—H5	0.9500
N1—H1A	0.901 (17)	C6—H6	0.9500
N1—H1B	0.887 (17)	C8—H8A	0.9800
C1—C6	1.3963 (17)	C8—H8B	0.9800
C1—C2	1.4038 (16)	C8—H8C	0.9800
C1—C7	1.5041 (16)		
			
C2—O1—C8	117.88 (9)	C3—C4—H4	119.8
C3—O2—H2	108.9 (12)	C6—C5—C4	119.98 (11)
C7—N1—H1A	118.7 (10)	C6—C5—H5	120.0
C7—N1—H1B	118.8 (10)	C4—C5—H5	120.0
H1A—N1—H1B	121.5 (14)	C5—C6—C1	120.95 (11)
C6—C1—C2	119.12 (10)	C5—C6—H6	119.5
C6—C1—C7	116.33 (10)	C1—C6—H6	119.5
C2—C1—C7	124.46 (11)	O3—C7—N1	120.85 (11)
O1—C2—C1	119.38 (10)	O3—C7—C1	119.49 (10)
O1—C2—C3	120.82 (10)	N1—C7—C1	119.65 (10)
C1—C2—C3	119.71 (11)	O1—C8—H8A	109.5
O2—C3—C4	122.52 (11)	O1—C8—H8B	109.5
O2—C3—C2	117.69 (10)	H8A—C8—H8B	109.5
C4—C3—C2	119.78 (11)	O1—C8—H8C	109.5
C5—C4—C3	120.36 (11)	H8A—C8—H8C	109.5
C5—C4—H4	119.8	H8B—C8—H8C	109.5
			
C8—O1—C2—C1	−129.31 (11)	O2—C3—C4—C5	177.18 (11)
C8—O1—C2—C3	54.02 (14)	C2—C3—C4—C5	−1.63 (18)
C6—C1—C2—O1	−173.41 (10)	C3—C4—C5—C6	2.32 (19)
C7—C1—C2—O1	10.24 (17)	C4—C5—C6—C1	−0.16 (19)
C6—C1—C2—C3	3.30 (17)	C2—C1—C6—C5	−2.65 (18)
C7—C1—C2—C3	−173.05 (10)	C7—C1—C6—C5	173.99 (11)
O1—C2—C3—O2	−3.41 (16)	C6—C1—C7—O3	−9.95 (17)
C1—C2—C3—O2	179.93 (10)	C2—C1—C7—O3	166.50 (11)
O1—C2—C3—C4	175.47 (10)	C6—C1—C7—N1	170.31 (11)
C1—C2—C3—C4	−1.20 (17)	C2—C1—C7—N1	−13.25 (18)

### Hydrogen-bond geometry (Å, º)

**Table:**

*D*—H···*A*	*D*—H	H···*A*	*D*···*A*	*D*—H···*A*
N1—H1*A*···O3^i^	0.901 (17)	2.056 (17)	2.9547 (13)	175.3 (14)
N1—H1*B*···O1	0.887 (17)	1.977 (17)	2.6789 (13)	135.0 (14)
O2—H2···O3^ii^	0.88 (2)	1.83 (2)	2.6895 (12)	165.5 (17)

Symmetry codes: (i) −*x*+1, −*y*+2, −*z*+1; (ii) *x*−1/2, −*y*+3/2, *z*+1/2.
